# Taurine reverses long-term memory deficits caused by LPS induced neuroinflammation

**DOI:** 10.1186/s12868-026-01017-2

**Published:** 2026-06-11

**Authors:** Maria Vaitsa Loch Haskel, Letícia Stresser da Silva Vequi, Marianna Wirthmann Pompeo Flauzino, Vinicius da Silva Correa, Cláudia Sirlene Oliveira, Meire Ellen Pereira, Elizama de Gregório, Juliana Sartori Bonini, Weber Claudio da Silva

**Affiliations:** 1 Postgraduate Program in Physiology, Federal University of Rio Grande do Sul, Porto Alegre, RS 90050-170 Brasil; 2 Laboratory of Neuropsychopharmacology, Department of Pharmacy, State University of Centre-West of Paraná, Guarapuava, PR 85040-167 Brasil; 3 Institute of Research Pelé Pequeno Príncipe, Curitiba, PR 80250-060 Brasil; 4 Faculties Pequeno Príncipe, Curitiba, PR 80230-020 Brasil

**Keywords:** Neurodegenerative diseases, Neuroinflammation, Taurine, Spatial long-term memory, Aversive memory

## Abstract

**Background:**

Despite neuroinflammation being an initially protective response made by the central nervous system (CNS), as it becomes chronic, it can lead to neuronal damage since the cytokines which are released by microglia potentialize cellular death due to excitotoxicity, and this, in turn, promotes the release of pro-inflammatory mediators, feeding this way a self-sustaining cycle of neuroinflammatory response, which favor subsequent neurodegeneration. Given the fact that until this moment, there are not any therapeutic alternatives able to stop the neurodegeneration, the objective of the present work was to evaluate the putative neuroprotector effect of taurine, a partial glycinergic ionotropic receptor agonist, and also a GABA_A_ receptor agonist, in a neuroinflammation animal model.

**Method:**

For this intent, the oral taurine administration was evaluated on mnemonic impairing caused by LPS induced neuroinflammation in male Wistar rats. Such effects were investigated on recent and late spatial long-term memory and aversive memory in the behavioural tasks Morris water maze (MWM) and context fear conditioning (CFC), respectively. In addition, we investigated the effect of orally administered taurine on hippocampal neuronal density and on hippocampal levels of TNF-α and IL-4.

**Results:**

Taurine, when orally administered for 30 days, in the doses of 20 and 200 mg/kg, was able to reverse the mnemonic impairment caused by neuroinflammation on recent and remote spatial long-term memory, and in the dose of 200 mg/kg, it also was able to do the same on aversive long-term memory. In the doses of 20 and 200 mg/kg, taurine was also able to reverse the LPS-induced increase in hippocampal TNF-α levels.

**Conclusion:**

Taurine, when orally administered in a pathological context characterized by neuroinflammatory background, as it was induced in this work, can perform a dose-dependent neuroprotective effect, probably by acting in an excitotoxic scenario in which the activation of hyperpolarizing receptors can be welcomed.

**Supplementary Information:**

The online version contains supplementary material available at 10.1186/s12868-026-01017-2.

## Introduction

Neurodegenerative diseases like Alzheimer’s, Parkinson’s, frontotemporal dementia, Huntington’s and others are common and increasing causes of morbidity, cognitive impairments, and mortality among elderly people. It is estimated that only Alzheimer’s Disease (AD) affects roughly 24 million people worldwide [1]. In addition to neurodegenerative diseases, other pathologies that affect the central nervous system (CNS), like cerebrovascular accidents, viral infections and paraneoplastic disorders, are also characterized by neuronal damage, which is often associated with chronic activation of the CNS innate immunologic response [2]. A self-sustaining chronic inflammatory environment is generated when these innate and adaptive immunologic mechanisms cannot properly combat the pathology triggering factors [3]. These mechanisms become one of the key factors contributing to neurodegeneration progression [4].

The neuroinflammatory process is characterized by microglia and astrocytes activation, inflammatory mediators in the parenchyma, and alterations in the brain-blood barrier, rendering the CNS susceptible to peripheric immunologic stimuli, like macrophage and T and B cells infiltration [5, 6, 4]. The microglia activation triggers pro-inflammatory cytokines secretion, like tumour necrosis factor-alpha (TNF-α) and interleukin 1 beta (IL-1b), as well as superoxide, and nitric oxide (NO), reactive oxygen species (ROS) and proteases [7, 8]. The cytokines TNF-α and IL-1b can interact with the glutamatergic system inducing an excitotoxicity state [5]. The glutamate-induced excitotoxicity leads to neuronal death in neurodegenerative diseases and acute conditions like infections. Just as pro-inflammatory cytokines can stimulate glutamatergic toxicity, this toxicity, by its time, can trigger the release of more pro-inflammatory mediators [5], feeding a vicious neurodegenerative circle. In this sense, hyperpolarizing drugs like glycinergic agonists can be an attractive therapeutic alternative with a potential neuroprotector effect [9].

So, considering that there are no efficient treatments for neurodegenerative diseases until this moment and that no therapeutic alternative can stop neurodegenerative progress [4], the present study aimed to evaluate the potential cognitive neuroprotector effect of a glycinergic and GABA_A_ agonist, taurine, in lipopolysaccharide (LPS) induced animal model of neuroinflammation. LPS is a gram-negative bacterial cell wall component, a potent inductor of immunological response, which has been largely used in neuroinflammation experimental models [10–14]. LPS acts mainly through its ligation to the receptor Toll-like 4 (TLR4). In the CNS, TLRs are expressed in neurons and glial cells, and when activated, they start a pro-inflammatory response, which can chronically lead to a neurodegenerative process [15].

## Materials and methods

### Ethics approval and consent to participate

All procedures were performed with investigators blinded to the animals’ treatment groups. The experiments complied with the guidelines of the National Institutes of Health for the care and use of laboratory animals and received approval from the Animal Care and Ethics Committee of the University of Center-West of Paraná (protocol numbers 029/2018 and 018/2019). In addition, all experimental procedures adhered to the ARRIVE guidelines (https://arriveguidelines.org) for reporting animal research.

### Subjects

Male Wistar rats, three months old and weighing between 220 and 280 g, obtained from the animal facility at the State University of Ponta Grossa and housed in our institutional animal facility, were used. The animals were housed in groups of four to five per cage and kept at a controlled temperature of 21–23 °C under a 12-hour light/dark cycle (lights on at 07:00), with unrestricted access to food and water.

### Experimental design

It was evaluated the oral administration of taurine effect on the LPS induced neuroinflammatory impairment of rats’ recent and late long-term memory retention. These rats underwent stereotaxic surgery for bilateral intra-hippocampal infusion of LPS or saline. After three days of recovery, they were orally administered with saline or taurine at doses of 2, 20 or 200 mg/kg for 30 days. These animals were weighed every 7 days, and their respective doses were adjusted according to their body weight. On the next day, after the end of taurine administration, the animals were trained in the Morris water maze task (MWM) for five consecutive days (8 trials per day). After the last training day, they were tested 24 h (recent long-term spatial memory) and 5 and 10 days (late long-term spatial memory). One day after the last test in the MWM task, they were trained in the contextual fear conditioning task (CFC) and tested for this task 24 h (recent long-term aversive memory) and 48 h (late long-term aversive memory) afterwards. Then, the animals were deeply anaesthetized with 75 mg/kg ketamine (König) plus 10 mg/kg xylazine (Coopers) and subsequently euthanized by decapitation. Their brains were then removed either for subsequent histological examination of the site of the lesion caused by stereotactic LPS infusion, or for hippocampi dissection for subsequent biochemical analysis. Only animals with the correct lesion site (hippocampal CA1) were included in the statistical analyses (94 out of 100 rats).

### Stereotaxic surgery for LPS infusion

To induce neuroinflammation, LPS was (from Escherichia coli 055:B5; Sigma) dissolved in saline at a concentration of 2,5 mg/ml. Rats were deeply anaesthetized with 75 mg/kg ketamine (König) plus 10 mg/kg xylazine (Coopers), and saline or 10 µg/side of LPS was bilaterally infused (4 µl for 10 min) into the pyramidal cell layer of the dorsal CA1 region, using coordinates (–4.2 anterior, ± 3.0 lateral, − 2.0 ventral from bregma) taken from the atlas of Paxinos and Watson [16]. The animals were allowed to recover from surgery for 3 d before submitting them for any other procedure.

### Drugs

LPS from Escherichia coli 055:B5 and taurine were purchased from Sigma and were first dissolved to the working concentration with saline and stored frozen at − 20 °C until use.

### Morris water maze task

The water maze consisted of a circular pool painted dark blue, measuring 180 cm in diameter, and conceptually divided into four equal quadrants for analytical purposes. The water was maintained at a temperature between 21 and 23 °C. A black circular platform (12 cm in diameter) was positioned 2 cm below the water surface, remaining invisible to the animals. Its rough texture enabled the rats to climb onto it easily once located.

The animals’ swimming trajectories were recorded by a video camera placed above the center of the pool and subsequently analyzed using a homemade computerized tracking system. The maze was situated in a brightly illuminated white room containing posters and other distal visual cues on the walls to aid spatial orientation.

Before the training phase, rats were handled for 5 min per day over three consecutive days. Training followed a spaced protocol conducted across five consecutive days [17–19]. Each day included eight training trials, during which the hidden platform remained in a fixed position. For each trial, the rats were released from different starting points and allowed to swim, followed by a 30-second stay on the platform. If an animal failed to locate the platform within 60 s, it was gently guided to it by the experimenter.

Memory retention was assessed through a 60-second probe trial without the platform, conducted 24 h, 5 days, or 10 days after the final training session.

### Contextual fear conditioning

The contextual fear conditioning task equipment (Med-Associates Inc., St. Albans, VT) consisted of a rectangular 25 × 32,2 × 25 cm box, with its central wall made of transparent plastic to allow the animal behaviour observation. The box floor is a grid made of 19 stainless steel bars, connected to an electric current generator, conducting a small intensity shock to animal paws when activated. This device is inside another chamber, measuring 75,5 × 63,5 × 35,8 cm, to isolate the inner box from noises, luminosity alterations and experimenter presence. A camera is attached inside this outer chamber, allowing animal behaviour to be observed. The training consisted of one unique session, in which each rat was put for 3 min inside the inner chamber for acclimatization and then received three 0,8 mA shocks in its paws, each shock during 3 s, 30 s between each shock. After the shocks, each rat stayed for more than 2 min in the inner chamber and then returned to its home cage. The tests were achieved 24 and 48 h after the training session. Each animal was put again inside the inner conditioning chamber for 5 min. The freezing (absence of body and head movement) test time percentage was registered as the aversive memory retention parameter [20]. The inner chamber was cleaned with alcohol 70% between each rat training and testing.

### Histological analysis

To investigate the effects of intra-hippocampal LPS and taurine treatment on neuronal integrity, histological analyses of the hippocampus were performed on brain sections stained with hematoxylin and eosin. Neuronal density was assessed in the CA1 region, allowing for the identification of possible morphological changes associated with the inflammatory process. Neuronal counts were expressed as the number of neurons per square millimeter (neurons/mm²).

### Cytokine analysis

Tumor necrosis factor (TNF-α) and Interleukin-4 (IL-4) levels in hippocampal tissue samples were determined by the Enzyme Linked Immuno Sorbent Assay (ELISA). For this, the samples were homogenized in 100mM sodium phosphate buffer, pH 7.0, centrifuged at 4 °C at 12.000 rpm for 15 min and the supernatant was aliquoted for analysis. After that, TNF-α and IL-4 levels were detected from the previously prepared sample using an ELISA kit (RAB0480 and RAB0302, Sigma-Aldrich^®^) according to the manufacturer’s instructions. The absorbance reading was performed by spectrophotometry at 450 nm and the results presented as pg/mg protein.

### Data analysis

The normality test Shapiro-Wilk was made across all groups at all time points, which were found to meet the assumptions of normality and homogeneity. The behavioural data are presented as mean and mean standard error. Data were analyzed by a two-tailed Student t-test or ANOVA followed by Dunnett’s post hoc tests (groups vs. naive).

## Results

### Effects of orally administered taurine on recent and late spatial long-term memory impairment caused by neuroinflammation

The animals were submitted to stereotaxic surgery for bilateral infusion of either saline or LPS in the 10 µg/side dose into the CA1 region of the dorsal hippocampus. After three days of surgery recovering, the animals from both groups (saline and LPS) were orally administered with saline or taurine in the doses of 2 mg/kg, 20 mg/kg or 200 mg/kg for 30 days, and, after the last day of treatment, they were trained in MWM for five days. Then these animals were tested in this task 24 h after the last diary training session to evaluate recent long-term memory, and then 5 and 10 afterwards to evaluate late long-term memory.

**Fig. 1:**
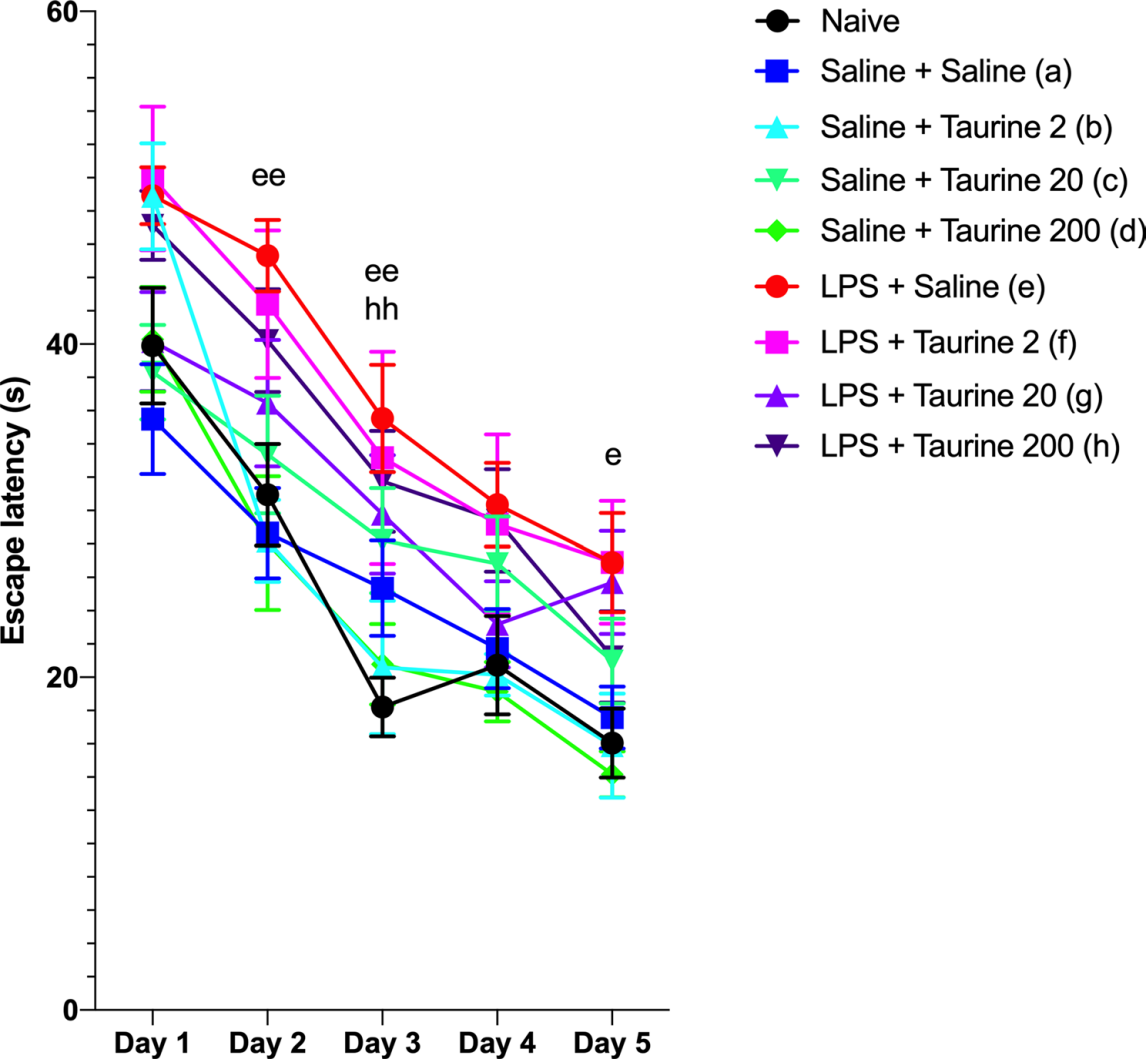
Taurine orally administered in the doses of 20 mg/kg and 200 mg/kg for 30 days reverse the impairment in retention of recent and remote long-term spatial memory caused by LPS induced hippocampal inflammation. Saline or LPS (10 µg/side) was bilaterally infused into CA1 region of male rats’ hippocampi. After this, these rats were orally treated with saline or taurine in the dose of 2, 20 or 200 mg/kg for 30 consecutive days. Then, the rats were trained for 5 days in Morris water maze task. Naïve: rats which did not go through stereotaxic surgery; S + S: intra-hippocampal (i.h.) saline + oral saline; S + T2, S+T20 or S+T200: i.h. saline + oral taurine 2, 20 or 200 mg/kg, respectively; LPS + S: i.h. LPS + oral saline; LPS+T2, LPS+T20 or LPS+T200: i.h. LPS + oral taurine 2, 20 or 200 mg/kg, respectively. Acquisition of spatial memory retention were measured as mean latency of 8 trials per daily session to find the hidden platform during each 60 s trial. Data are expressed as mean ± mean error. e *P* < 0.05, ee *P* < 0.01 of LPS + S vs. Naïve group, hh *P* < 0.01 of LPS+T200 vs. Naïve group, two-way ANOVA with repeated measures (daily sessions): F_Training day_(3.72, 320) = 139.3, F_Treatment_(8, 86) = 3.995, F_Interaction_(32, 344) = 1.579, Dunnett’s multiple comparisons test (within each row, compare columns, simple effects within rows); *n* = 6–16 per group

Initially, we verified that the rats learned the task over the training days, which was evidenced by the reduction in the latency (time between the start of the trial and the localization of the platform by a given rat) average time during the training (two-way ANOVA with repeated measures, F_Training day_ (3.72, 320) = 139.3, *p* < 0.0001), as shown in Fig. 1. There were significative difference mainly between Naïve and LPS + Saline group (F_treatment_ (8, 86) = 3.995, *p* = 0.0005).

**Fig. 2:**
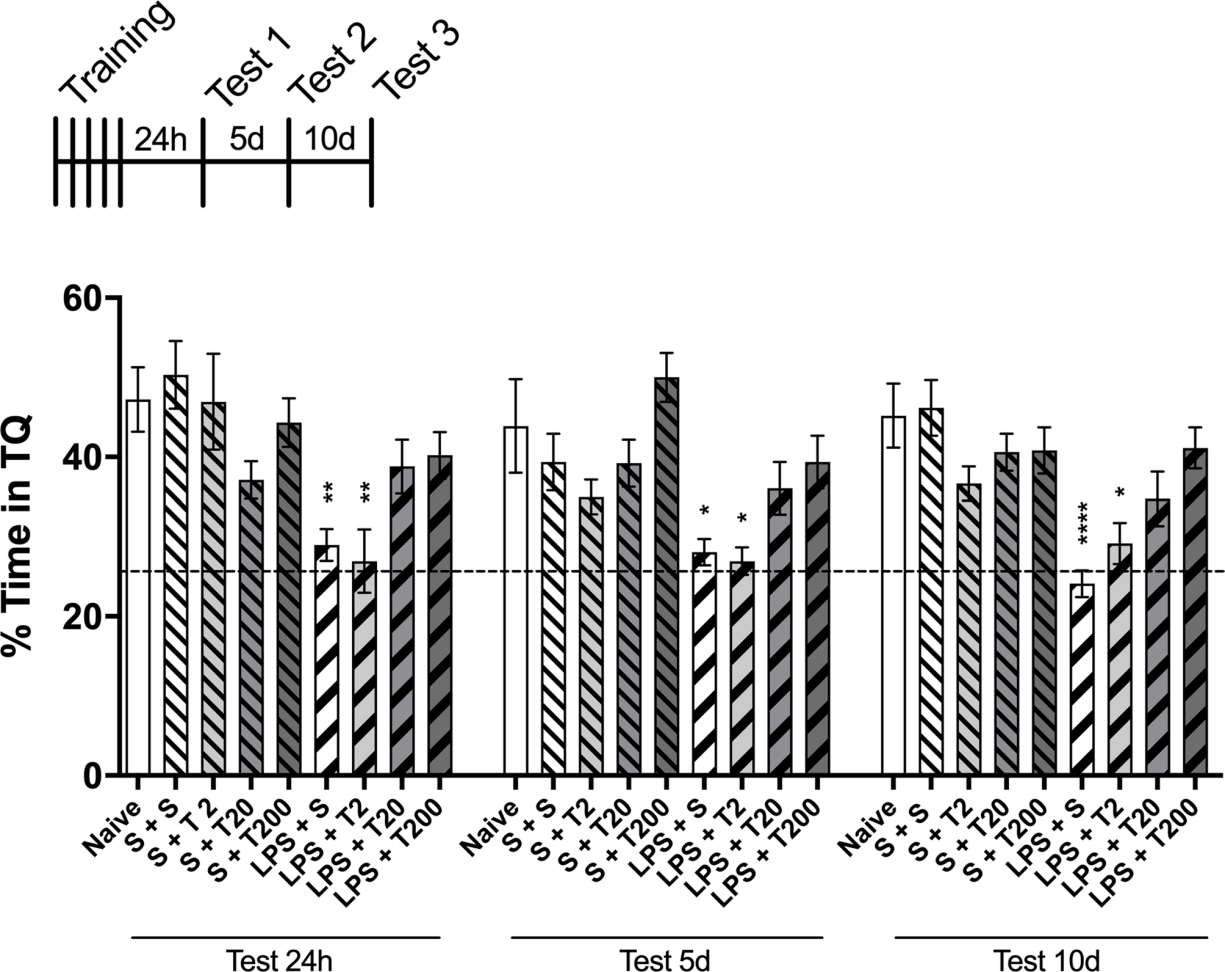
Taurine orally administered in the doses of 20 mg/kg and 200 mg/kg for 30 days reverse the impairment in retention of recent and remote long-term spatial memory caused by LPS induced hippocampal inflammation. Saline or LPS (10 µg/side) was bilaterally infused into CA1 region of male rats’ hippocampi. After this, these rats were orally treated with saline or taurine in the dose of 2, 20 or 200 mg/kg for 30 consecutive days. Then, the rats were trained for 5 days in Morris water maze task, and tested 24 h, 5 and 10 days after the last training day. Naïve: rats which did not go through stereotaxic surgery; S + S: intra-hippocampal (i.h.) saline + oral saline; S + T2, S+T20 or S+T200: i.h. saline + oral taurine 2, 20 or 200 mg/kg, respectively; LPS + S: i.h. LPS + oral saline; LPS+T2, LPS+T20 or LPS+T200: i.h. LPS + oral taurine 2, 20 or 200 mg/kg, respectively. Recent and remote spatial memory retention were measured as the percentage of time spent inside target quadrant during 60 s of each test. Data are expressed as mean ± mean error. *P < 0.05, **P < 0.01 and ****P < 0.0001 vs. respective Naïve group, one-way ANOVA: F(8, 86) = 4.810, 3.940 and 6.052 for tests 24 h, 5 and 10d post-training, respectively, Dunnett’s post test; *n* = 6–16 per group

There was no retention of either recent or late mnemonic trace in the groups LPS + Saline (LPS + S) and LPS + Taurine 2 mg/kg (LPS + T2) since the rats in these groups did not show in the tests a performance significantly different of the theoretical mean of 25% of test time searching the target quadrant, as expected for aleatory swimming over the pool quadrants. Also, we verified that the target quadrant searching in these groups were significantly less than that observed in the naïve group (test 24 h post-training: one-way ANOVA, F_(8, 86)_ = 4.810, *p* < 0.01 in Dunnett post-test for both groups LPS + S and LPS + T2; test 5d post-training: one-way ANOVA, F_(8, 86)_ = 3.949, *p* < 0.05 in Dunnett post-test for both groups LPS + S and LPS + T2; test 10d post-training: one-way ANOVA, F_(8, 86)_ = 6.052, *p* < 0.0001 and *p* < 0.05 in Dunnett post-test respectively for the groups LPS + S and LPS + T2). On the other hand, there were neither recent nor late long-term spatial memory retention impairment in the groups LPS + Taurine 20 mg/kg (LPS + T20) and LPS + Taurine 200 mg/kg (LPS + T200), indicating that taurine was able to revert the mnemonic deficit caused by previous LPS-induced hippocampal neuroinflammation in the oral doses of 20 and 200 mg/kg for 30 days (Fig. 2).

### Effects of orally administered taurine on aversive long-term memory impairment caused by neuroinflammation

After we have verified that orally administered taurine for 30 days could revert the mnemonic impairment caused by LPS induced neuroinflammation on recent and late long-term spatial memory retention, as shown before, we investigated if a similar effect also occurs in another sort of memory.

**Fig. 3:**
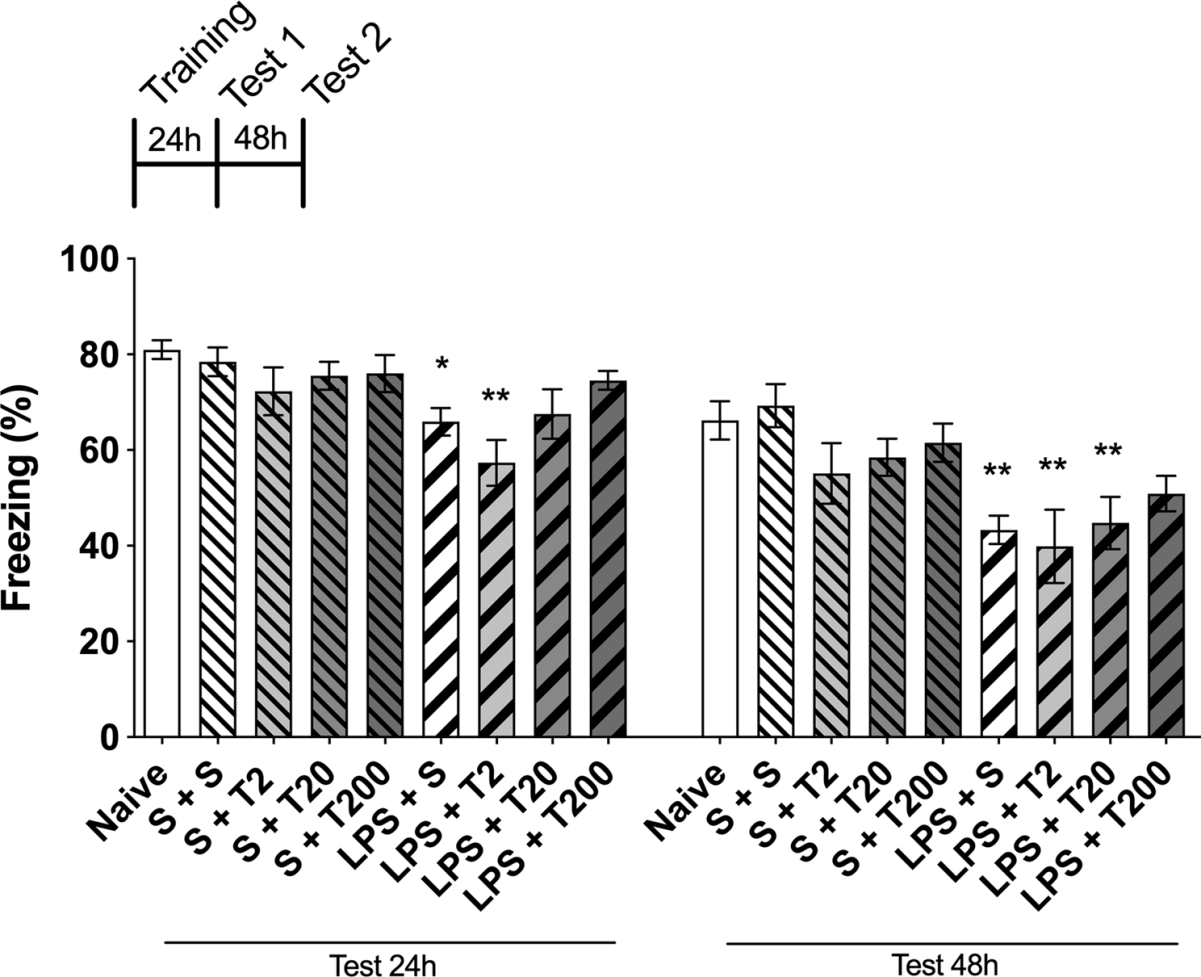
Taurine orally administered in the doses of 20 mg/kg and 200 mg/kg for 30 days reverse the impairment in retention of recent long-term spatial memory caused by LPS induced hippocampal inflammation, but only taurine in the dose of 200 mg/kg has the same effect on the impairment in retention of remote long-term spatial memory. Saline or LPS (10 µg/side) was bilaterally infused into CA1 region of male rats’ hippocampi. After this, these rats were orally treated with saline or taurine in the dose of 2, 20 or 200 mg/kg for 30 consecutive days. Then, the rats were trained in the contextual fear conditioning task, and tested 24 and 48 h post-training. Naive: rats which did not go through stereotaxic surgery; S + S: intra-hippocampal (i.h.) saline + oral saline; S + T2, S+T20 or S+T200: i.h. saline + oral taurine 2, 20 or 200 mg/kg, respectively; LPS + S: i.h. LPS + oral saline; LPS+T2, LPS+T20 or LPS+T200: i.h. LPS + oral taurine 2, 20 or 200 mg/kg, respectively. Recent and remote aversive memory retention were measured as the percentage of time freezing during 5 min of each test. Data are expressed as mean ± mean error. *p < 0.05; **p < 0.01 vs. respective Naïve group, one-way ANOVA: F(8, 86) = 3.225 and 4.779 for tests 24 h and 48 post-training, respectively, Dunnett’s post test; *n* = 6–16 per group

Therefore, one day after the last test in the MWM, the animals were submitted to a unique training session in freezing conditioning task and tested 24 h and 48 h afterwards. There was significantly lower retention of recent mnemonic trace in the groups LPS + Saline (LPS + S) and LPS + Taurine 2 mg/kg (LPS + T2), and also in the group LPS + Taurine 20 mg/kg (LPS + T20) for late mnemonic trace, since the rats in these groups showed in the tests a relative freezing time significantly worse than naïve group (test 24 h post-training: one way ANOVA, F_(8, 86)_ = 3.225, *p* < 0.05 and *p* < 0.01 respectively in Dunnett post-test for groups LPS + S and LPS + T2; test 48 h post-training: one way ANOVA, F_(8, 86)_ = 34.779, *p* < 0.01 in Dunnett post-test for groups LPS + S, LPS + T2 and LPS + T20). On the other hand, there were neither recent nor remote recent long-term aversive memory retention impairment in the group LPS + Taurine 200 mg/kg (LPS + T200), indicating that taurine was able to revert the mnemonic deficit caused by previous LPS-induced hippocampal neuroinflammation in the oral doses of 200 mg/kg for 30 days (Fig. 3).

### Effects of orally administered taurine and neuroinflammation on hippocampal neuronal density

**Fig. 4:**
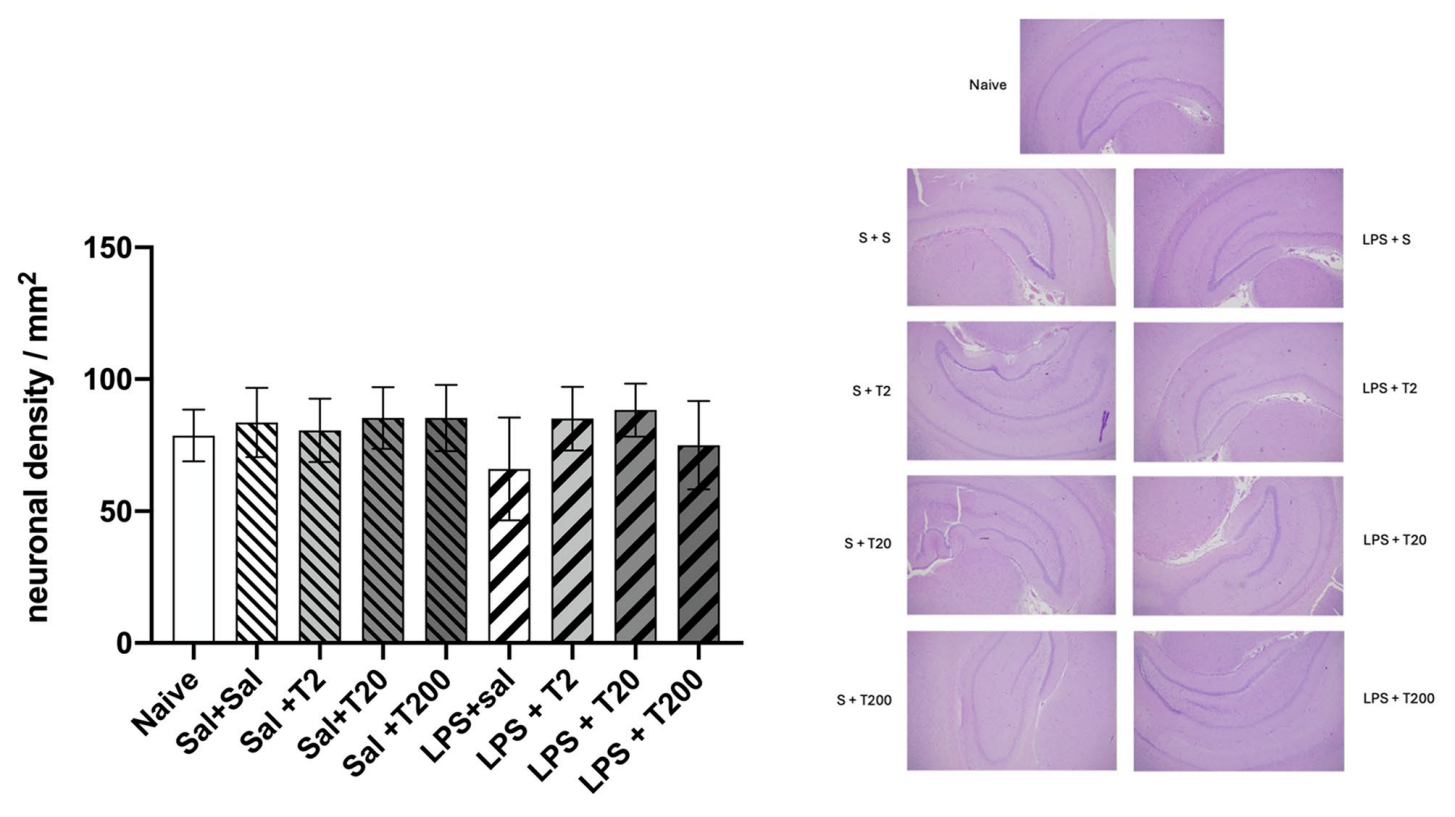
Neither intrahippocampal CA1 LPS (10 µg / side) nor taurine orally administered in the doses of 2, 20 or 200 mg/kg for 30 days affects neuronal integrity. Naive: rats which did not go through stereotaxic surgery; S + S: intra-hippocampal (i.h.) saline + oral saline; S + T2, S+T20 or S+T200: i.h. saline + oral taurine 2, 20 or 200 mg/kg, respectively; LPS + S: i.h. LPS + oral saline; LPS+T2, LPS+T20 or LPS+T200: i.h. LPS + oral taurine 2, 20 or 200 mg/kg, respectively. Data are expressed as mean ± mean error. *n* = 3 per group

In the CA1 region, there was no statistically significant difference between the groups and the naïve group (Fig. 4). Neuronal integrity was therefore preserved in all groups.

### Effects of orally administered taurine and neuroinflammation on hippocampal cytokine levels

**Fig. 5:**
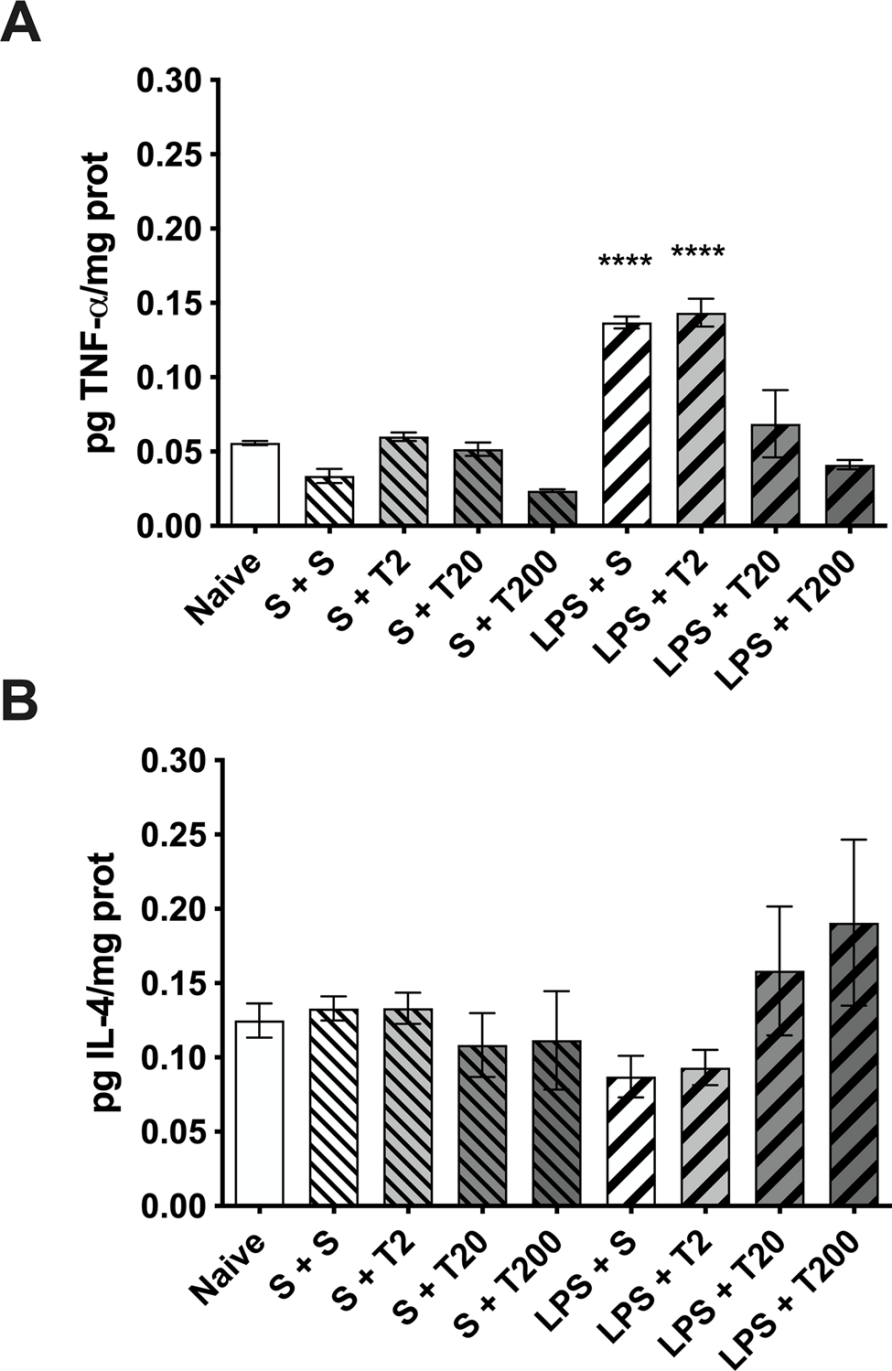
Intrahippocampal LPS increases TNF-α levels. Saline or LPS (10 µg/side) was bilaterally infused into CA1 region of male rats’ hippocampi. After this, these rats were orally treated with saline or taurine in the dose of 2, 20 or 200 mg/kg for 30 consecutive days. Then, the rats were trained either on the Morris water maze or the fear conditioning tasks. After completing these tasks, the rats were euthanized, their brains were removed, and their hippocampi were dissected for analysis of cytokine levels. Naive: rats which did not go through stereotaxic surgery; S + S: intra-hippocampal (i.h.) saline + oral saline; S + T2, S+T20 or S+T200: i.h. saline + oral taurine 2, 20 or 200 mg/kg, respectively; LPS + S: i.h. LPS + oral saline; LPS+T2, LPS+T20 or LPS+T200: i.h. LPS + oral taurine 2, 20 or 200 mg/kg, respectively. (**A**) TNF-α levels were statistically higher in the LPS + S and LPS + T2 groups than in the naive group. (**B**) There was no statistically significant difference in IL-4 levels compared to the naive group. ****p < 0.0001 vs. respective Naïve group, one-way ANOVA: F(8, 18) = 24.46, Dunnett’s post test; *n* = 3 per group

TNF-α and IL-4 levels were analyzed in hippocampal homogenates from the rats. The LPS + Saline (LPS + S) and LPS + Taurine 2 mg/kg (LPS + T2) groups exhibited significantly higher TNF-α levels than the naive group (one-way ANOVA, F_(8, 18)_ = 24.46, *p* < 0.0001 in the Dunnett post-hoc test for the LPS + S and LPS + T2 groups) (Fig. 5A). On the other hand, none of the groups differed significantly from the naive group in terms of IL-4 levels (Fig. 5B).

## Discussion

Several compounds have shown promise in preclinical models of cognitive impairment. For example, apomorphine has demonstrated memory-enhancing and neuroprotective effects in rat models of dementia [21]. However, the search for safe, orally bioavailable agents with favourable safety profiles continues. Taurine, an endogenous amino acid, represents a particularly attractive candidate due to its established safety in humans.

We verified that taurine in the oral doses of 20 and 200 mg/kg administered for 30 days was able to revert the impairment caused by intra-hippocampal infusion of LPS on retention of recent and remote spatial and aversive long-term memory as respectively evaluated in the tasks Morris water maze and contextual fear conditioning. In addition, taurine at the same doses of 20 and 200 mg/kg was also able to reduce TNF-α levels in the hippocampus of rats. On the other hand, neither IL-4 levels nor neuronal density were altered, whether by intra-hippocampal LPS or by taurine itself.

It is well known that dietary and nutraceutical interventions can modulate memory through interactions with neurotransmitter pathways [22]. So, a putative explanation for these results is that taurine, a glycinergic ionotropic agonist [23] and GABA_A_ ionotropic receptor partial agonist [24], promotes anionic current influx into the cell, hyperpolarizing it, hampering its depolarization and thus reducing calcium influx into the cell, which in turn inhibits excitotoxic mechanisms triggered by excessive NMDA receptors and L-VDCC activation in pathological situations involving neuroinflammatory processes [25] similar to that was experimentally induced in the present work.

To reinforce this assumption, previous studies have already demonstrated that taurine binding to GABA_A_ receptor indeed reduces the excessive activation of NMDA receptors [26, 27]. Additionally, considering that GABAergic neurotransmission is downgraded by ageing due to diverse factors, like GABA concentration and its receptor functionality reduction, and that this inhibitory function reduction can alter many cognitive functions, it was shown that taurine chronically administrated for 8 months improved memory acquisition and retention in aged mice [28]. So, these findings suggest that taurine supplementation can slow down the GABAergic function decline that occurs in ageing.

Taurine also has important functions in brain development, and its concentrations are reduced with age [29, 30]. For example, in cortical neuron culture from newborn rats, taurine promotes neurites growth and stimulates synapses formation [31]. In another experiment with cells culture [32], in an encephalic trauma model, neurons and astrocytes were treated with taurine at 100, 200, and 300 mg/l for 72 h. The results showed that taurine was able to reduce reactive oxygen species in the damaged cells, decreasing lipidic peroxidation, increasing reduced glutathione (GSH) concentration, and the activity of superoxide dismutase (SOD), catalase, glutathione peroxidase and acetylcholinesterase (AChE), this way exercising antioxidant action. In the same study, it was observed that taurine treatment exercised anti-inflammatory action by decreasing the levels of TNF-α and IL-6 and decreasing apoptotic signals such as caspase-3, p53, bcl-2 and bax.

Taurine had no effect on its own, as observed in the results for the S + T20 and S + T200 groups, whether in behavioral experiments, neuronal density, or hippocampal TNF-α levels. This lack of effect of taurine alone indicates that its ability to reverse the effects of intra-hippocampal LPS infusion supports a neuroprotective rather than a cognitive-enhancing interpretation of these results.

It is also important to highlight that taurine does not cross the blood-brain barrier (BBB) easily [33]. A Na^+^ and Cl^−^ dependent transport system can be regulated by many factors, for instance, osmolarity and TNF-α. In rats, it was seen that, after TNF-α brain microinjection, there is a decrease in brain taurine efflux to the blood, inversely proportional to TNF-α concentration [34]. Moreover, it was observed in an in vitro BBB model (brain capillary endothelial cells culture), that the TNF-α treatment increased the absorption of taurine by the cells, as well as the TauT (taurine transporter) messenger RNA expression [35], showing that TNF-α modulates taurine transport across BBB. Once TNF-α is a pro-inflammatory cytokine released in large quantities by astrocytes and microglia facing pathological situations [3], and consistent with our findings showing that there was an increase in TNF-α levels in the LPS + S and LPS + T2 groups, such evidence strengthens our putative explanation that taurine exercises neuroprotective action when orally administered in a neuroinflammatory context by binding GABA_A_ and glycine receptors since TNF-α releasing increases in an inflammatory state. This cytokine modulates taurine crossing through BBB, as discussed above. There is also the possibility that, in future studies, we may use nanostructured formulations of taurine in order to ensure greater penetration of the blood-brain barrier, thereby enabling us to achieve the same results with lower doses [36].

Taurine also prevents glutamatergic excitotoxicity by regulating intracellular [Ca^2+^] and the energetic mitochondrial metabolism. Mitochondria has a large cytoplasmic calcium capture capacity, and when glutamate receptors are excessively stimulated, they promote a large calcium influx into the cell, overcharging energetic mitochondrial metabolism. In cell culture, previous treatment with taurine promoted the intracellular calcium basal levels restoration, thereupon the glutamate receptors activation, then showing taurine role in regulating calcium homeostasis [37].

Finally, our results suggest that further preclinical experiments should be conducted that mimic a chronic inflammatory condition rather than an acute one, as presented in this study, in order to support appropriate preclinical-to-clinical translation steps.

## Conclusion

Based on these results, we can conclude that taurine, when orally administered for 30 days in the doses of 20 mg/kg and 200 mg/kg, was able to reverse the mnemonic impairment caused by LPS induced neuroinflammation in rats’ recent and late long-term spatial memory, as well as recent long-term aversive memory. At these same doses, taurine was also able to reverse the increase in hippocampal TNF-α levels. In contrast, only taurine in the dose of 200 mg/kg was able to do the same on late aversive long-term memory.

## Supplementary Information

Below is the link to the electronic supplementary material.

Supplementary Material 1

## Data Availability

The raw data is available as supplementary material named “Dataset.doc”.

## Declarations

### Additional information

Correspondence and requests for materials should be addressed to W.C.S.
