# Supplementary material for: Taurine reverses long-term memory deficits caused by LPS induced neuroinflammation
Source: BMC Neurosci. 2026 Jun 11;27:30. doi: 10.1186/s12868-026-01017-2 (PMC13483688; doi:10.1186/s12868-026-01017-2)
Supplement: Supplementary file 1 [file 12868_2026_1017_MOESM1_ESM.docx]

**Dataset: Raw Data**

**Figure 1**

Naive group

| Training day | Rat  #1 | Rat  #2 | Rat  #3 | Rat  #4 | Rat  #5 | Rat #6 | Rat  #7 | Rat  #8 | Rat  #9 |
| --- | --- | --- | --- | --- | --- | --- | --- | --- | --- |
| Day 1 | 38.375^a^ | 24.250 | 38.375 | 43.000 | 43.625 | 39.875 | 25.625 | 48.000 | 58.125 |
| Day 2 | 16.125 | 22.625 | 37.375 | 29.875 | 46.000 | 30.000 | 23.625 | 36.125 | 36.750 |
| Day 3 | 9.875 | 13.750 | 17.625 | 16.250 | 21.125 | 24.500 | 15.125 | 18.750 | 26.875 |
| Day 4 | 16.500 | 25.000 | 35.250 | 10.250 | 30.500 | 21.375 | 7.625 | 20.875 | 19.125 |
| Day 5 | 10.375 | 11.375 | 14.500 | 13.000 | 29.125 | 22.375 | 13.250 | 11.875 | 18.500 |

a: Each cell contains a mean of 8 latency trials per rat per day session, in seconds.

Saline + Saline

| Training day | Rat  #1 | Rat  #2 | Rat  #3 | Rat  #4 | Rat  #5 | Rat  #6 | Rat  #7 | Rat  #8 | Rat  #9 | Rat #10 |
| --- | --- | --- | --- | --- | --- | --- | --- | --- | --- | --- |
| Day 1 | 43.750 | 39.250 | 37.375 | 34.625 | 27.625 | 32.875 | 25.500 | 25.250 | 59.625 | 29.125 |
| Day 2 | 17.750 | 26.875 | 36.000 | 47.625 | 31.375 | 20.625 | 22.250 | 26.625 | 30.750 | 26.625 |
| Day 3 | 15.125 | 33.250 | 33.250 | 39.125 | 14.500 | 33.125 | 24.250 | 14.125 | 25.125 | 21.625 |
| Day 4 | 13.625 | 34.000 | 15.750 | 22.125 | 17.125 | 26.875 | 21.500 | 11.125 | 31.625 | 23.500 |
| Day 5 | 11.875 | 21.500 | 23.375 | 20.875 | 12.375 | 18.375 | 23.875 | 6.125 | 21.875 | 15.625 |

Saline + Taurine 2

| Training day | Rat  #1 | Rat  #2 | Rat  #3 | Rat  #4 | Rat  #5 | Rat  #6 |
| --- | --- | --- | --- | --- | --- | --- |
| Day 1 | 44.625 | 51.750 | 45.250 | 55.125 | 37.625 | 58.875 |
| Day 2 | 30.250 | 33.500 | 28.875 | 34.000 | 18.375 | 24.125 |
| Day 3 | 24.250 | 32.625 | 16.250 | 29.875 | 12.375 | 8.125 |
| Day 4 | 18.875 | 20.250 | 20.125 | 22.500 | 15.125 | 24.000 |
| Day 5 | 24.250 | 24.250 | 7.375 | 19.250 | 11.750 | 8.500 |

Saline + Taurine 20

| Training day | Rat  #1 | Rat  #2 | Rat  #3 | Rat  #4 | Rat  #5 | Rat  #6 |
| --- | --- | --- | --- | --- | --- | --- |
| Day 1 | 33.375 | 27.250 | 43.125 | 26.625 | 50.875 | 45.625 |
| Day 2 | 25.000 | 29.125 | 57.875 | 16.625 | 34.375 | 37.125 |
| Day 3 | 15.500 | 23.250 | 39.875 | 14.250 | 23.750 | 48.125 |
| Day 4 | 18.750 | 23.500 | 48.375 | 18.500 | 26.750 | 35.250 |
| Day 5 | 23.375 | 14.375 | 40.000 | 13.875 | 31.625 | 19.000 |
| Training day | Rat  #7 | Rat  #8 | Rat  #9 | Rat  #10 | Rat  #11 |  |
| Day 1 | 39.500 | 32.875 | 37.125 | 30.000 | 55.000 |  |
| Day 2 | 39.125 | 18.875 | 37.125 | 28.000 | 43.750 |  |
| Day 3 | 30.250 | 30.500 | 25.750 | 21.000 | 38.125 |  |
| Day 4 | 26.500 | 16.625 | 27.625 | 19.875 | 33.375 |  |
| Day 5 | 22.000 | 9.875 | 17.125 | 20.375 | 19.000 |  |

Saline + Taurine 200

| Training day | Rat  #1 | Rat  #2 | Rat  #3 | Rat  #4 | Rat  #5 | Rat  #6 | Rat  #7 | Rat  #8 | Rat  #9 | Rat #10 |
| --- | --- | --- | --- | --- | --- | --- | --- | --- | --- | --- |
| Day 1 | 35.625 | 23.125 | 34.750 | 59.375 | 44.000 | 46.250 | 34.500 | 39.250 | 49.500 | 36.625 |
| Day 2 | 12.125 | 13.750 | 18.250 | 48.625 | 20.250 | 45.000 | 24.375 | 35.250 | 26.750 | 36.250 |
| Day 3 | 7.375 | 22.000 | 32.000 | 27.375 | 20.625 | 21.125 | 10.625 | 25.625 | 15.375 | 25.625 |
| Day 4 | 14.625 | 16.125 | 23.375 | 20.375 | 17.625 | 31.375 | 13.000 | 14.375 | 16.750 | 23.750 |
| Day 5 | 12.625 | 16.125 | 17.375 | 17.000 | 12.500 | 17.125 | 5.125 | 10.125 | 13.250 | 20.375 |

LPS + Saline

| Training day | Rat  #1 | Rat  #2 | Rat  #3 | Rat  #4 | Rat  #5 | Rat  #6 | Rat  #7 |
| --- | --- | --- | --- | --- | --- | --- | --- |
| Day 1 | 60.000 | 40.875 | 50.625 | 49.375 | 50.625 | 51.875 | 40.625 |
| Day 2 | 58.125 | 50.375 | 48.375 | 46.500 | 44.125 | 34.250 | 41.375 |
| Day 3 | 38.250 | 44.750 | 24.125 | 24.125 | 33.000 | 26.875 | 19.000 |
| Day 4 | 25.875 | 31.500 | 21.375 | 36.125 | 23.875 | 12.375 | 21.750 |
| Day 5 | 44.875 | 18.250 | 17.375 | 21.625 | 43.125 | 12.375 | 23.375 |
| Training day | Rat  #8 | Rat  #9 | Rat  #10 | Rat  #11 | Rat  #12 | Rat  #13 |  |
| Day 1 | 41.750 | 50.500 | 56.125 | 54.500 | 42.000 | 47.125 |  |
| Day 2 | 39.250 | 59.750 | 49.125 | 42.125 | 39.125 | 36.625 |  |
| Day 3 | 41.625 | 58.125 | 36.500 | 41.625 | 49.625 | 24.375 |  |
| Day 4 | 29.625 | 44.125 | 35.875 | 43.125 | 36.000 | 32.875 |  |
| Day 5 | 36.000 | 29.625 | 14.375 | 23.250 | 28.000 | 37.250 |  |

LPS + Taurine 2

| Training day | Rat  #1 | Rat  #2 | Rat  #3 | Rat  #4 | Rat  #5 | Rat  #6 |
| --- | --- | --- | --- | --- | --- | --- |
| Day 1 | 46.500 | 53.250 | 53.375 | 30.250 | 57.125 | 59.250 |
| Day 2 | 54.625 | 37.000 | 24.375 | 42.625 | 44.125 | 51.625 |
| Day 3 | 41.625 | 20.500 | 25.375 | 18.875 | 32.625 | 60.000 |
| Day 4 | 22.625 | 22.250 | 23.875 | 19.250 | 32.625 | 54.500 |
| Day 5 | 18.500 | 22.750 | 25.125 | 20.750 | 31.250 | 43.000 |

LPS + Taurine 20

| Training day | Rat  #1 | Rat  #2 | Rat  #3 | Rat  #4 | Rat  #5 | Rat  #6 | Rat  #7 |
| --- | --- | --- | --- | --- | --- | --- | --- |
| Day 1 | 55.000 | 45.875 | 26.375 | 33.250 | 32.625 | 37.250 | 47.875 |
| Day 2 | 60.000 | 36.250 | 22.875 | 31.375 | 44.000 | 40.000 | 47.000 |
| Day 3 | 50.250 | 35.625 | 15.875 | 18.625 | 19.500 | 36.750 | 27.750 |
| Day 4 | 30.125 | 30.125 | 14.875 | 22.375 | 10.000 | 20.500 | 25.625 |
| Day 5 | 34.000 | 51.750 | 22.750 | 23.500 | 20.625 | 30.000 | 30.750 |
| Training day | Rat  #8 | Rat  #9 | Rat  #10 | Rat  #11 | Rat  #12 | Rat  #13 |  |
| Day 1 | 26.375 | 48.750 | 37.625 | 56.000 | 52.500 | 22.375 | 40.375 |
| Day 2 | 17.000 | 47.375 | 25.375 | 59.750 | 33.375 | 31.625 | 14.250 |
| Day 3 | 14.750 | 39.000 | 25.375 | 59.750 | 29.500 | 18.375 | 25.750 |
| Day 4 | 21.500 | 30.000 | 12.500 | 37.750 | 40.500 | 10.125 | 18.500 |
| Day 5 | 14.500 | 40.250 | 9.250 | 18.625 | 32.000 | 13.625 | 18.125 |

LPS + Taurine 200

| Training day | Rat  #1 | Rat  #2 | Rat  #3 | Rat  #4 | Rat  #5 | Rat  #6 | Rat  #7 | Rat  #8 |
| --- | --- | --- | --- | --- | --- | --- | --- | --- |
| Day 1 | 38.875 | 46.500 | 39.500 | 46.500 | 60.000 | 60.000 | 43.750 | 58.000 |
| Day 2 | 27.625 | 40.625 | 24.375 | 50.375 | 48.750 | 49.750 | 21.625 | 59.875 |
| Day 3 | 18.125 | 16.500 | 21.000 | 22.250 | 35.375 | 53.875 | 41.750 | 56.750 |
| Day 4 | 23.500 | 19.000 | 34.875 | 12.250 | 42.250 | 33.750 | 33.625 | 47.250 |
| Day 5 | 21.375 | 13.375 | 12.250 | 15.125 | 42.875 | 36.500 | 11.875 | 25.875 |
| Training day | Rat  #9 | Rat  #10 | Rat  #11 | Rat  #12 | Rat  #13 | Rat  #14 | Rat  #15 | Rat  #16 |
| Day 1 | 47.500 | 50.375 | 44.875 | 60.000 | 41.875 | 39.250 | 34.875 | 42.335 |
| Day 2 | 48.500 | 42.750 | 36.125 | 52.875 | 46.000 | 43.000 | 16.250 | 34.791 |
| Day 3 | 29.375 | 24.250 | 25.875 | 44.500 | 36.750 | 25.500 | 25.625 | 30.627 |
| Day 4 | 19.000 | 28.125 | 23.500 | 60.000 | 25.250 | 18.125 | 21.625 | 28.478 |
| Day 5 | 11.625 | 20.375 | 14.125 | 41.875 | 29.375 | 19.750 | 10.750 | 12.526 |

**Figure 2**

Test 24h post-training (% Time in TQ)

| Naive | S + S | S + T2 | S + T20 | S + T200 | LPS + S | LPS + T2 | LPS + T20 | LPS + T200 |
| --- | --- | --- | --- | --- | --- | --- | --- | --- |
| 53.33 | 56.67 | 28.33 | 43.33 | 55.00 | 26.67 | 36.67 | 46.67 | 43.33 |
| 45.00 | 48.33 | 50.00 | 30.00 | 53.33 | 26.67 | 13.33 | 33.33 | 18.33 |
| 73.33 | 66.67 | 40.00 | 36.67 | 38.33 | 16.67 | 28.33 | 26.67 | 66.67 |
| 45.00 | 48.33 | 71.67 | 40.00 | 43.33 | 23.33 | 35.00 | 41.67 | 31.67 |
| 35.00 | 48.33 | 51.67 | 35.00 | 28.33 | 31.67 | 16.67 | 65.00 | 50.00 |
| 31.67 | 31.67 | 40.00 | 53.33 | 36.67 | 33.33 | 31.67 | 48.33 | 45.00 |
| 51.67 | 48.33 |  | 30.00 | 51.67 | 38.33 |  | 60.00 | 28.33 |
| 41.67 | 48.33 |  | 28.33 | 35.00 | 28.33 |  | 33.33 | 40.00 |
| 48.33 | 31.67 |  | 36.67 | 45.00 | 41.67 |  | 26.67 | 46.67 |
|  | 75.00 |  | 45.00 | 56.67 | 21.67 |  | 41.67 | 51.67 |
|  |  |  | 30.00 |  | 36.67 |  | 25.00 | 43.33 |
|  |  |  |  |  | 30.00 |  | 30.00 | 41.67 |
|  |  |  |  |  | 21.67 |  | 38.33 | 23.33 |
|  |  |  |  |  |  |  | 26.67 | 41.67 |
|  |  |  |  |  |  |  |  | 33.33 |
|  |  |  |  |  |  |  |  | 38.33 |

Test 5d post-training (% Time in TQ)

| Naive | S + S | S + T2 | S + T20 | S + T200 | LPS + S | LPS + T2 | LPS + T20 | LPS + T200 |
| --- | --- | --- | --- | --- | --- | --- | --- | --- |
| 30.00 | 40.00 | 33.33 | 41.67 | 50.00 | 23.33 | 23.33 | 43.33 | 53.33 |
| 55.00 | 40.00 | 35.00 | 45.00 | 63.33 | 30.00 | 26.67 | 40.00 | 20.00 |
| 30.00 | 35.00 | 31.67 | 53.33 | 48.33 | 20.00 | 33.33 | 11.67 | 46.67 |
| 73.33 | 63.33 | 43.33 | 31.67 | 51.67 | 31.67 | 26.67 | 43.33 | 50.00 |
| 28.33 | 33.33 | 38.33 | 31.67 | 68.33 | 31.67 | 30.00 | 61.67 | 38.33 |
| 33.33 | 50.00 | 28.33 | 46.67 | 45.00 | 33.33 | 21.67 | 33.33 | 33.33 |
| 28.33 | 30.00 |  | 41.67 | 51.67 | 30.00 |  | 48.33 | 30.00 |
| 51.67 | 31.67 |  | 31.67 | 45.00 | 33.33 |  | 30.00 | 58.33 |
| 65.00 | 25.00 |  | 50.00 | 40.00 | 33.33 |  | 26.67 | 35.00 |
|  | 45.00 |  | 38.33 | 36.67 | 28.33 |  | 30.00 | 41.67 |
|  |  |  | 20.00 |  | 16.67 |  | 26.67 | 30.00 |
|  |  |  |  |  | 20.00 |  | 48.33 | 26.67 |
|  |  |  |  |  | 33.33 |  | 33.33 | 20.00 |
|  |  |  |  |  |  |  | 28.33 | 36.67 |
|  |  |  |  |  |  |  |  | 66.67 |
|  |  |  |  |  |  |  |  | 43.33 |

Test 10d post-training (% Time in TQ)

| Naive | S + S | S + T2 | S + T20 | S + T200 | LPS + S | LPS + T2 | LPS + T20 | LPS + T200 |
| --- | --- | --- | --- | --- | --- | --- | --- | --- |
| 41.67 | 50.00 | 33.33 | 40.00 | 40.00 | 13.33 | 20.00 | 38.33 | 46.67 |
| 46.67 | 45.00 | 36.67 | 35.00 | 63.33 | 26.67 | 33.33 | 28.33 | 26.67 |
| 65.00 | 38.33 | 45.00 | 48.33 | 30.00 | 23.33 | 31.67 | 20.00 | 41.67 |
| 61.67 | 45.00 | 30.00 | 40.00 | 40.00 | 18.33 | 36.67 | 18.33 | 41.67 |
| 33.33 | 31.67 | 40.00 | 43.33 | 45.00 | 36.67 | 23.33 | 40.00 | 43.33 |
| 36.67 | 43.33 | 35.00 | 53.33 | 43.33 | 21.67 | 30.00 | 55.00 | 46.67 |
| 51.67 | 58.33 |  | 35.00 | 40.00 | 21.67 |  | 35.00 | 25.00 |
| 38.33 | 30.00 |  | 31.67 | 33.33 | 30.00 |  | 10.00 | 45.00 |
| 31.67 | 63.33 |  | 31.67 | 33.33 | 28.33 |  | 35.00 | 51.67 |
|  | 56.67 |  | 51.67 | 40.00 | 21.67 |  | 31.67 | 45.00 |
|  |  |  | 36.67 |  | 18.33 |  | 33.33 | 45.00 |
|  |  |  |  |  | 26.67 |  | 40.00 | 25.00 |
|  |  |  |  |  | 26.67 |  | 51.67 | 30.00 |
|  |  |  |  |  |  |  | 50.00 | 41.67 |
|  |  |  |  |  |  |  |  | 63.33 |
|  |  |  |  |  |  |  |  | 40.00 |

**Figure 3**

Test 24h post-training (%Time of freezing)

| Naive | S + S | S + T2 | S + T20 | S + T200 | LPS + S | LPS + T2 | LPS + T20 | LPS + T200 |
| --- | --- | --- | --- | --- | --- | --- | --- | --- |
| 79.00 | 92.33 | 67.67 | 59.00 | 89.33 | 65.00 | 53.67 | 20.33 | 72.67 |
| 75.00 | 83.33 | 78.33 | 70.33 | 73.33 | 78.00 | 47.67 | 80.00 | 81.00 |
| 88.67 | 73.00 | 53.00 | 81.33 | 82.00 | 62.33 | 69.33 | 86.00 | 78.33 |
| 72.67 | 75.67 | 67.67 | 79.67 | 52.33 | 45.00 | 64.00 | 88.00 | 71.67 |
| 83.00 | 82.67 | 79.00 | 77.67 | 69.33 | 72.67 | 68.33 | 55.67 | 89.00 |
| 81.00 | 71.00 | 88.00 | 89.67 | 66.67 | 70.00 | 41.00 | 60.33 | 69.00 |
| 75.67 | 66.67 |  | 69.00 | 70.33 | 64.33 |  | 65.00 | 88.67 |
| 89.00 | 95.00 |  | 76.67 | 94.33 | 48.00 |  | 63.67 | 73.00 |
| 85.00 | 70.67 |  | 87.33 | 84.33 | 78.00 |  | 43.33 | 72.33 |
|  | 74.33 |  | 62.00 | 78.33 | 74.67 |  | 76.67 | 70.67 |
|  |  |  | 78.00 |  | 73.67 |  | 79.67 | 74.67 |
|  |  |  |  |  | 63.00 |  | 79.33 | 60.33 |
|  |  |  |  |  | 62.67 |  | 90.00 | 66.00 |
|  |  |  |  |  |  |  | 57.67 | 67.33 |
|  |  |  |  |  |  |  |  | 75.67 |
|  |  |  |  |  |  |  |  | 83.00 |

Test 48h post-training (%Time of freezing)

| Naive | S + S | S + T2 | S + T20 | S + T200 | LPS + S | LPS + T2 | LPS + T20 | LPS + T200 |
| --- | --- | --- | --- | --- | --- | --- | --- | --- |
| 87.67 | 77.00 | 47.33 | 51.33 | 75.67 | 25.33 | 37.33 | 11.67 | 35.00 |
| 52.33 | 70.33 | 63.00 | 51.00 | 56.33 | 28.67 | 24.67 | 57.00 | 41.00 |
| 69.33 | 60.33 | 51.33 | 58.33 | 60.33 | 33.00 | 25.33 | 54.33 | 69.33 |
| 56.33 | 68.00 | 36.33 | 67.33 | 47.67 | 43.00 | 51.00 | 68.00 | 62.00 |
| 68.00 | 54.00 | 81.67 | 59.67 | 56.00 | 46.00 | 72.33 | 35.00 | 64.00 |
| 80.67 | 89.33 | 51.00 | 89.00 | 37.67 | 52.33 | 28.67 | 26.00 | 40.00 |
| 59.67 | 72.67 |  | 49.67 | 79.00 | 53.00 |  | 40.00 | 52.00 |
| 67.00 | 92.33 |  | 55.33 | 69.67 | 38.67 |  | 48.33 | 47.00 |
| 55.00 | 49.00 |  | 62.33 | 68.33 | 51.33 |  | 11.00 | 37.67 |
|  | 60.00 |  | 37.67 | 65.00 | 55.67 |  | 67.00 | 90.00 |
|  |  |  | 61.67 |  | 57.00 |  | 48.67 | 56.67 |
|  |  |  |  |  | 34.00 |  | 54.67 | 41.00 |
|  |  |  |  |  | 45.33 |  | 77.33 | 48.00 |
|  |  |  |  |  |  |  | 27.67 | 35.33 |
|  |  |  |  |  |  |  |  | 53.67 |
|  |  |  |  |  |  |  |  | 41.67 |

**Figure 4**

Neuronal density / mm^2^

| Naive | S + S | S + T2 | S + T20 | S + T200 | LPS + S | LPS + T2 | LPS + T20 | LPS + T200 |
| --- | --- | --- | --- | --- | --- | --- | --- | --- |
| 81 | 69 | 67 | 72 | 73 | 88 | 85 | 98 | 56 |
| 68 | 94 | 89 | 91 | 85 | 51 | 73 | 89 | 87 |
| 87 | 88 | 86 | 93 | 98 | 59 | 97 | 78 | 82 |

**Figure 5**

pg TNF-α/mg prot

| Naive | S + S | S + T2 | S + T20 | S + T200 | LPS + S | LPS + T2 | LPS + T20 | LPS + T200 |
| --- | --- | --- | --- | --- | --- | --- | --- | --- |
| 0,058 | 0,041453 | 0,054938 | 0,05973874 | 0,022694 | 0,14478219 | 0,1267267 | 0,108307 | 0,034828 |
| 0,053503 | 0,025193 | 0,065011 | 0,043875 | 0,023104 | 0,132522 | 0,159364 | 0,029958 | 0,0440488 |
| 0,055834 | 0,034267 | 0,06025 | 0,051352 | 0,025374 | 0,133173 | 0,144391 | 0,067679 | 0,0440488 |

pg IL-4/mg prot

| Naive | S + S | S + T2 | S + T20 | S + T200 | LPS + S | LPS + T2 | LPS + T20 | LPS + T200 |
| --- | --- | --- | --- | --- | --- | --- | --- | --- |
| 0,124259 | 0,139585 | 0,116057 | 0,1473398 | 0,174766 | 0,062166 | 0,114058 | 0,214259 | 0,081563 |
| 0,105144 | 0,116843 | 0,131012 | 0,073406 | 0,063257 | 0,11077 | 0,072654 | 0,073004 | 0,265754 |
| 0,145236 | 0,142571 | 0,152133 | 0,104311 | 0,096513 | 0,088376 | 0,092935 | 0,187529 | 0,224137 |
